# Dynamic Evolution, Regional Differences, and Spatial Spillover Effects of Urban Ecological Welfare Performance in China from the Perspective of Ecological Value

**DOI:** 10.3390/ijerph192316271

**Published:** 2022-12-05

**Authors:** Jun Wang, Guixiang Zhang

**Affiliations:** 1 College of Urban Economics and Public Administration, Capital University of Economics and Business, Beijing 100070, China; 2 Beijing Key Laboratory of Megaregions Sustainable Development Simulation, Capital University of Economics and Business, Beijing 100070, China

**Keywords:** urban ecological welfare performance, sustainable economic development, ecological value, regional difference, spatial spillover effect

## Abstract

Ecological welfare performance (EWP) is a necessary condition for achieving sustainable economic development and is a crucial initiative for resolving the dilemma of balancing economic development, social welfare, ecology, and the environment. This paper constructs and enhances a comprehensive evaluation system of ecological welfare performance (EWP) from an ecological value viewpoint for the purpose of making the results of the evaluation both comprehensive and objective. In the meantime, the Dagum Gini decomposition, kernel density, and the spatial Durbin model were initiated to measure and analyze urban EWP, which supplies new empirical results for studies on the dynamic evolution, regional differences and driving factors of urban EWP. The findings indicate the following: (1) In each spatial dimension, the urban EWP roughly demonstrates first a decreased and then an increased trend. There is a discrepancy in the east–central–west distribution of urban EWP in space, in which urban EWP in the east and west is larger than that in the central area. (2) For relative differences, intra-regional and inter-regional differences in urban EWP are significantly spatially uneven. Supervariable density is the main source of regional differences. For absolute differences, the EWP demonstrates a significant polarization effect. (3) The urban EWP does not have σ-convergence; nonetheless, it has spatial absolute *β*-convergence and spatial conditional *β*-convergence. (4) The urban EWP has a significant spatial correlation. Industrial structure, science and technology innovation, foreign investment, urbanization, government intervention, finance development, and environmental regulations all have influence effects and spatial effects on urban EWP; notwithstanding, the direction and magnitude of the effects vary across the different spatial dimensions.

## 1. Introduction

Improving social welfare and enhancing residents’ well-being is the fundamental purpose of economic and social development [1]. At present, the connotation of sustainable development is gradually changing from the two-dimensional perspective of “economic growth and green ecology” to the integrated dimension of “economic growth, green ecology, and social welfare” [2]. Since the reform and opening up, China’s economy has achieved a high level of success and is now the second-largest economy in the world, and social welfare has been continuously improved. However, for a long time, the crude economic development model has achieved rapid growth in economic speed but accumulated more problems in ecosystem destruction and environmental pollution. These problems have become a major bottleneck limiting sustainable economic development and social welfare enhancement [3]. Therefore, how to enhance economic growth and social welfare within the ecological and environmental carrying capacity and promote the coordinated development of the economy, society, ecology, and environment are the major opportunities and challenges to achieve the goal of high-quality development at this stage. The EWP refers to the efficiency of using ecological resources as inputs in exchange for economic benefits and social welfare [4]. Improving EWP not only reflects the concept of sustainable development, but it is also a concrete measure to realize the high-quality development pattern of “lucid waters and lush mountains are invaluable assets.”. In the eastern, central, and western regions of China, cities differ greatly in the economic base, resource endowment, technological innovation, and industrial structure, which inevitably leads to spatial differences in urban EWP and is not conducive to the formation of the coordinated development pattern among regions. Based on this, this paper analyses the spatiotemporal evolution and regional differences of urban EWP among the national, eastern, central, and western regions; measures sources of differences and convergence characteristics; and explores influential mechanisms and realization routes. This is significant for promoting the balanced development of EWP among regions; enhancing the coordinated development of economy, society, ecology, and environment; and accelerating the realization of sustainable economic development.

Neoclassical economics assumes that the economy can grow indefinitely and that social welfare will continue to rise alongside it [5]. This view was considered correct for a long time after the beginning of the Industrial Revolution and is widely practiced in actual social production [6]. During this period, ecological resources were seen as natural capital with unlimited access, and a large number of resources and energy were consumed in the process of achieving economic growth. However, man-made capital is considered a scarce resource and a leading force for economic growth because of the high concentration of technology and human resources [7]. However, with the rapid progress of industrialization around the world, more and more problems of environmental pollution, ecosystem destruction, and resource and energy depletion have been exposed, and the increase in economic speed can no longer rely entirely on the past crude development model [8]. The integrity of ecosystems, the degree of environmental pollution, and the number of resources and energy reserves have gradually become the main limiting factors for economic development and social welfare enhancement, and ecological capital has become a scarce resource. EWP, as an efficiency indicator that measures the conversion of unit resource factor inputs into economic growth and social welfare enhancement, has become an important way to measure the sustainable development of regional economies and has received progressive attention from society and academia. With the introduction of high-quality development, the government and society advocate the economic and social development approach of “lucid waters and lush mountains are invaluable assets.” [9], and EWP gradually becomes one of the important indicators to measure the level of synergistic development of economic development, social welfare, ecological safety, and environmental protection.

The evaluation of sustainable development mainly adopts the way of constructing the indicator system, but there are two shortcomings. On the one hand, in order to pursue the completeness and comprehensiveness of the measurement system, the indicator system has become increasingly large, but this weakens the influence of the core indicators. On the other hand, the selection of specific measurement indicators mostly relies on the researcher’s experience, which leads to a strong subjectivity of the indicator system [10]. From the input–output perspective, EWP emphasizes the efficiency of the transformation from ecological resource inputs to welfare outputs. At present, under the condition of the absolute scarcity of natural resources, it is a fundamental requirement for sustainable development to enhance the conversion efficiency of resource consumption within the ecological carrying capacity. EWP reflects the achievement of sustainable economic development and the enhancement of people’s sense of ecological gain under the premise of ensuring the quality of the ecological environment. It can more precisely and objectively represent the deviations of the complex system of cities in the process of sustainable development. Therefore, EWP is an effective tool for studying regional sustainable development [11].

The concepts related to ecological welfare include the following. (1) Social welfare and ecological welfare. The concept of ecological welfare is formed by combining ecology and welfare under the notion of ecologism. Between the pursuit of unlimited economic growth and the opposition to it, ecological welfare offers a path to the sustainable development of the economy and society [4]. It highlights the significance and value of ecological quality to human life and gives an ecological connotation to welfare. Currently, with the emergence of ecological crises and environmental degradation events, ecological welfare has become an important indicator of social welfare, with sustainable development and quality of life as the core objectives for its enhancement [12]. (2) Well-being and welfare. Well-being refers to the psychological reflection of a person’s needs being met, namely, happiness and satisfaction. It is a micro-level subjective consciousness that cannot be precisely measured [12]. Welfare refers to the utility, benefit, or objective thing that produces happiness, and it belongs to the macro category. It can be measured by objective indicators. (3) Ecological welfare and ecological well-being. Ecological well-being refers to the subjective sense of happiness obtained from ecosystems and the environment [7]. Ecological welfare refers to the improvement of human society’s production and lifestyle through the use of objective material forms such as energy and resources obtained from nature, thereby enhancing welfare. It includes sustainable economic development, effective pollution control, and improvement of quality of life [1]. Maintaining an effective ecosystem cycle is key to the continuous improvement of ecological welfare [11]. Based on this, ecological welfare performance refers to the efficiency of translating ecological inputs into objective welfare, while ecological well-being performance is the efficiency of translating into the happiness of residents.

With the increasing attention of government and society on EWP, the study about EWP has been conducted at a more in-depth stage. At present, the research results on EWP can be broadly divided into three aspects.

First, the definition and measurement index system and the measurement method. For the definition and measurement index, Daly first proposed that, based on economic and ecological dimensions, EWP mainly refers to the degree of social welfare enhancement in exchange for the unit consumption of resources and energy, but did not explicitly propose a feasible measurement method [13]. As an output indicator, the Human Development Index (HDI) is proposed to provide a possibility for measuring EWP. As a widely used measure standard, HDI enriches the connotation of EWP from three aspects of residents’ health, years of education, and survivability [14]. Input indicators are mostly measured by the ecological footprint [15], but this measure does not fully reflect the market value of ecological resources. With the rise of sustainable development economics, the view that ecological factors should be included in the economic accounting system has gradually gained recognition. As a result, a few studies have begun to include ecological value as an input factor in the construction of input indicators [16]. The specific measures of EWP can be divided into two categories. One category is based on Daly’s view and uses the ratio method to measure it [17]. The ratio method cannot take multiple indicators into consideration and measurement due to its singularity characteristic, which leads to a lack of comprehensiveness and objectivity. The other category is based on a comprehensive multi-indicator measurement system using stochastic frontier analysis (SFA) [18] and a data envelopment model (DEA) [19]. Data envelopment models are more widely used because of the various model types and the ability to analyze EWP including non-desired outputs.

Second, the analysis of EWP’s regional differences and evolutionary features. Most of the available studies focus on a static analysis of regional differences. Du et al. (2019) measured and analyzed the regional variability characteristics of EWP in the eastern, central, and western regions of China by using the Theil index [20]. A few studies have begun to focus on the dynamic evolutionary features of differences and their sources, but less literature considers both absolute and relative differences. Guo et al. (2022) used the Dagum Gini decomposition model to measure EWP’s intra-regional differences, inter-regional differences, and sources of differences in the Yangtze River Economic Zone, finding that the spatial heterogeneity of EWP was prominent and inter-regional differences were the main source of overall regional differences [21]. Based on the province dimension, Guo et al. (2022) measured EWP and analyzed the characteristics of regional relative differences and regional absolute differences by the Dagum Gini coefficient and kernel density estimation methods [2]. For convergence analysis, σ-convergence and *β*-convergence are mostly used for the analysis of EWP, but less research has considered the influence of spatial factors on the convergence characteristics. Deng et al. (2021) measured the EWP index of provincial regions in China from 2004 to 2017, and then analyzed the convergence characteristics by σ convergence, traditional and spatial *β* convergence models [22].

Third, the analysis of driving factors and influential mechanisms. The EWP is a multidimensional and comprehensive concept and its impact mechanisms are still in the exploration stage. The factors involved in the available studies include economic development, openness, environmental regulation, industrial structure, technological innovation, and government finance. Zhu and Zhang (2014) explored the relationship between EWP and economic growth based on 124 countries and regions, and the results showed that they have an inverted U-shaped relationship [23]. Deng et al., (2021) found that openness, environmental regulation, and industrial structure all have a facilitating effect on the improvement of EWP, while economic development has an inhibiting effect, and technology is not significant [22]. Guo et al., (2022) measured the relationship among fiscal vertical imbalance, environmental regulation, and EWP in Chinese provinces from 2005 to 2020, and found that government vertical imbalance has a suppressive effect on EWP, while environmental regulation shows a positive effect [24].

Regarding the spatial scale of the study, most of the existing studies take countries, regions, and provinces [2] as the subjects of study. Zhu and Zhang (2014) analyzed EWP based on more than 100 countries as the research spatial scale [23]. Xiao and Xiao (2021) explored the differentiation and spatial convergence of EWP from the perspective of the Yellow River Basin [16]. However, EWP at the microscopic spatial scale has not been studied in sufficient depth. China’s provinces have disparate differences in area, resource endowments, natural conditions, and economic and social development conditions. If the province is used as the study unit, it can only reflect the average level of cities within the province. This weakly characterizes the variability and heterogeneity of EWP within provinces. Cities are spatial carriers for promoting ecological civilization, important growth poles for social and economic development, and important subjects for achieving green development. Cities are complex systems that combine ecological, social, economic, service, and innovative functions. Therefore, studying the EWP at the urban scale is conducive to promoting coordinated development among regions and advancing the process of high-quality economic development.

Based on this, this paper further investigates the EWP in the following aspects. First, improving the indicator system. In most EWP indicator measurement systems, the input indexes are mainly resource consumption, and the output indicators are mainly the HDI [23,25]. With the proposal of high-quality development, this paper optimizes the indicator measurement system by adding ecological capital input and non-desired output. This makes the indicator system more consistent with the current development status, and the measurement results are more comprehensive and objective. Second, while some studies take provincial regions as the spatial scale [26], this paper analyzes EWP from the city dimension to more carefully portray spatial evolution. It also analyzes the dynamic evolution of the EWP’s regional differences based on relative differences and absolute differences. Third, the spatial factor has been less considered in the mechanisms of EWP in existing studies [27]. To reduce endogeneity errors, this paper explores the influential mechanisms and spatial effects of EWP by constructing the spatial econometric model.

Therefore, this paper first measures the EWP of 287 cities in China from 2000 to 2020 using the Super-SBM-DEA model that includes non-desired outputs. Second, based on the Dagum Gini coefficient decomposition and convergence analysis, this paper analyses the regional differences and convergence characteristics of urban EWP in different space dimensions. Then, spatial factors are incorporated into the mechanism analysis to explore the urban EWP’s driving factors by spatial econometric models. Finally, countermeasures and recommendations are proposed based on the findings of the study.

## 2. Measurement System, Research Methods, and Data Sources

### 2.1. Constructing the Measurement System

The coordinated development between environmental protection and economic development is not only the internal requirement for achieving high-quality development, but also the value goal of building a harmonious coexistence between man and nature [28]. The city is an important carrier of regional development. The urban EWP as well as the communication and interaction between cities directly determine the region’s comprehensive competitiveness and sustainable development. Therefore, the urban EWP measurement system should fully consider the essence and connotation of ecological civilization [18,25]. Specifically, the input indicators consist of two dimensions: resource consumption and ecological capital. The output indicators are divided into two dimensions: non-desired output and desired output (Table 1).

#### 2.1.1. Input Indicators

Most studies only take resource consumption and environmental pollution as input indicators, but this method ignores the market values of ecological resources [29,30]. Neoclassical growth theory believes that the natural environment is the material source of economic and social development, but the ecological value is not included in the economic accounting system [6]. This view directly leads to a crude economic development pattern, including the unlimited exploitation of natural resources and uncontrolled discharge of pollutants into the environment. Therefore, ecological services, as one of the necessary conditions for achieving sustainable economic and social development [31], should be included in the input indicator system in addition to resource consumption. Ecological capital is the sum of ecological factors that enter directly into the economic system of human society, participate in the process of social production and reproduction in the form of production factors, and create wealth and value together with other capital. It specifically includes the stock of ecological resources, the quality of the ecological environment, and the overall usefulness of ecosystem services [18]. Based on this, it can be divided into three types, namely, ecological resource capital, ecological environment capital, and ecological service capital. Ecological resource capital refers to a category of ecological capital that exists in the state of ecological resources, including tangible and intangible resources such as energy and minerals. Ecological environment capital is a kind of ecological capital used to maintain the objective environmental state. It can be optimized in a variety of ways, including investments in emission reduction in waste gas, wastewater, and solid waste, pollution control equipment, and environmental protection services. Ecological service capital refers to ecological capital that can meet people’s ecological consumption needs and improve the pleasure and happiness of life, including investment in nature conservation and ecological construction, and investment in ecological infrastructure construction that improves urban comfort. In this paper, ecological resource capital is mainly captured by the indicator “resource consumption”. Ecological environment capital is mainly reflected by the “ecological environment improvement” indicator. Ecological service capital is represented by the “ecological service supply” indicator. Most of the available studies only use “resource consumption” as an input indicator. This paper further incorporates the other two types of ecological capital into “ecological capital” to enrich the input indicators. For the sake of the continuity of the research results, “resource consumption” and “ecological capital” are included as the one-level indicators. Based on the availability of data, ecological capital is measured from two aspects: ecological environment improvement and ecological service supply.

In resource consumption, water consumption is expressed in terms of water resource per capita, namely, total urban water resources divided by the corresponding population size. Due to missing data, this paper calculates the total water resources of each city separately based on the total water resources of the province, with the ratio of urban population to the corresponding province population as a reference.

#### 2.1.2. Output Indicators

Output indicators are measured by desired and undesired outputs. In existing studies, social welfare has mainly been considered in terms of population health, education level, and the ability to live a decent life [32]. However, this measurement ignores the importance of ecology and the environment. Good ecology and environment are the most universal well-being factors for people’s livelihoods, and, in order to meet people’s needs for a better life, we must achieve coordination between economic development and environmental protection [33]. In particular, the non-desired output indicators of pollutant emissions are added to measure the green development and environmental management of cities. The desired output indicators are based on the dimensions of the human development index, including residents’ health, culture and education, and economic capacity. However, the indicators of expected output are supplemented and refined for data availability and measurement comprehensiveness. Due to the lack of data on average life expectancy over a long time series, the population health is measured by the number of doctors and hospital beds. Culture and educational attainment are measured by the number of library collections, teachers, and students. Economic capacity, in addition to the GDP per capita, also includes the savings per capita and fiscal budget per capita.

#### 2.1.3. Driving Factors

The improvement of EWP is influenced by a combination of factors. (1) Industrial structure. The key to improving economic development quality lies in building a modern industrial system [34]. By optimizing the industrial structure and promoting industrial transformation and upgrading, we can reduce resource consumption and pollutant emissions and promote environmental quality. (2) Technological innovation. Innovation is the endogenous driving force for economic development in the new era. The science and technology innovation-driven strategy helps improve enterprise production technology, increase total factor social productivity, reduce pollutant emissions, promote the intensive use of resources and energy, and improve economic development quality [35]. (3) Foreign investment. Foreign investment is conducive to accelerating domestic industrial restructuring and optimization and improving production efficiency. We can take advantage of mature foreign technology and management systems to optimize traditional industries and promote high-tech industries and service industries. However, it also has disadvantages such as technological dependence, crowding out space for independent innovation, and pollution transfer with industry [36]. (4) Urbanization. The promotion of urbanization is conducive to the agglomeration effect and scale effect of various production factors, reducing intermediate costs, and promoting economic development [37]. It can also provide labor resources for industrial and tertiary industry, and effectively solves the employment problem [38]. (5) Government intervention. The market failure of ecological value and ecological services is the main reason why ecological resources are difficult to include in the economic accounting system [39,40]. Government intervention can compensate for the lack of market by fostering a unified ecological and environmental market [41]. However, government intervention can also increase the financial burden and decrease the flexibility and autonomy of the market and enterprises. (6) Finance development. Finance development and finance reform play an important role in promoting economic structural transformation and industrial iteration [42,43]. (7) Environmental regulation. The coordinated development of the economy and ecology cannot be achieved without the government’s formulation and implementation of reasonable environmental regulation policies [44].

Therefore, the following indicators are selected to analyze the influential mechanism of urban EWP. (1) Industrial structure (indus) is measured by the ratio of secondary industry output to GDP. (2) Science and technology innovation (tech) is expressed as the ratio of tech input to GDP. (3) This study uses the ratio of FDI to GDP as the proxy for foreign investment (fore). (4) Urbanization (urban) is expressed by the urbanization rate of the population. (5) Government intervention (govern) is measured by the ratio of fiscal budget expenditure to GDP. (6) This paper adopts the ratio of deposits to GDP to measure finance development (finan). (7) Environmental regulation (envir) is expressed by the ratio of the frequency of environment-related words in the government work reports.

### 2.2. Research Methods

#### 2.2.1. Super-SBM-DEA Model including Non-Desired Outputs

Based on the variable scale payoffs, output orientation, and undesired outputs, urban EWP is measured by the Super-SBM data envelopment model. This model can further differentiate decision units that are on the efficient frontier and include non-desired output indicators. It is also not necessary to set the model form in advance [4].

(1) $$\min E=\frac{\frac{1}{m}\sum_{i=1}^{m}\frac{\bar{x}}{x_{ik}}}{\frac{1}{s_{1}+s_{2}}\left(\sum_{u=1}^{s_{1}}\frac{\bar{y^{e}}}{y_{uk}^{e}}+\sum_{\tau =1}^{s^{2}}\frac{\bar{y^{w}}}{y_{\tau l}^{w}}\right)}$$

(2) $$s.t.\;\overset{n}{\underset{j=1,j\neq k}{\sum }}x_{ij}\lambda_{j}\leq x_{ij}$$

(3) $$\overset{n}{\underset{j=1,j\neq k}{\sum }}y_{rj}\lambda_{j}+s_{r}^{+}=y_{rk}$$

(4) $$\overset{n}{\underset{j=1,j\neq k}{\sum }}\lambda_{j}=1$$

(5) $$\lambda ,\;s^{-},s^{+}\geq 0$$

i=1,2,⋯,m;r=1,2,⋯,q;j=1,2,⋯,n(j≠k)

where *E* represents EWP; *k* is the decision unit number; *x*, *y_s_*, and *y_w_* represent input, desired output, and non-desired output; *m*, *u*, and *τ* are the corresponding quantities; *s^−^* and *s^+^* denote input slack and output slack variables; and *λ* represents the weight vector.

#### 2.2.2. Dagum Gini Coefficient Decomposition Model

The Dagum Gini coefficient decomposition model is often used to analyze regional differences and spatial imbalance characteristics [45]. Regional differences (*G*) in EWP can be divided into three components, including intra-regional difference contribution (*G_w_*), inter-regional difference contribution (*G_nb_*), and supervariable density (*G_t_*).

(6) $$G=\frac{\sum_{j=1}^{n}\sum_{h=1}^{n}\sum_{i=1}^{k_{j}}\sum_{r=1}^{k_{h}}\left|E_{ji}-E_{hr}\right|}{2E^{\prime }k^{2}}$$

(7) $$G_{w}=\overset{n}{\underset{j=1}{\sum }}G_{jj}p_{j}s_{j}$$

(8) $$G_{kb}=\overset{n}{\underset{j=2}{\sum }}\overset{j-1}{\underset{h=1}{\sum }}G_{jh}\left(p_{j}s_{h}+p_{h}s_{j}\right)D_{jh}$$

(9) $$G_{t}=\overset{n}{\underset{j=2}{\sum }}\overset{j-1}{\underset{h=1}{\sum }}G_{jh}\left(p_{j}s_{h}+p_{h}s_{j}\right)\left(1-D_{jh}\right)$$

where *n* and *k* represent the number of regions and cities; *E’* denotes the mean EWP; *p_j_* = *k_j_*/*k* is the ratio of the number of cities in region *j* to the total number of cities. *G_jj_*, *G_jh_*, and *D_jh_* are the Gini coefficient of region *j*, the Gini coefficient between region *j* and *h*, and the interaction effect on EWP between regions.

(10) $$G_{jj}=\frac{1}{2\bar{E_{j}}}\overset{k_{j}}{\underset{i=1}{\sum }}\overset{k_{j}}{\underset{r=1}{\sum }}\left|E_{ji}-E_{jr}\right|/k_{j}^{2}$$

(11) $$G_{jh}=\overset{k_{j}}{\underset{i=1}{\sum }}\overset{k_{h}}{\underset{r=1}{\sum }}\left|E_{ji}-E_{jr}\right|/k_{j}k_{h}\left(\bar{E_{j}}+\bar{E_{h}}\right)$$

(12) $$D_{jh}=\left(d_{jh}-p_{jh}\right)/\left(d_{jh}+p_{jh}\right)$$

(13) $$d_{jh}=\int_{0}^{\infty }dF_{j}\left(E\right)\int_{0}^{E}\left(E-x\right)dF_{h}\left(x\right)$$

(14) $$p_{jh}=\int_{0}^{\infty }dF_{h}\left(E\right)\int_{0}^{E}\left(E-x\right)dF_{j}\left(x\right)$$

The equations from (12) to (14) represent the EWP’s difference between regions, hypervariable first-order moments, and cumulative density distribution functions.

#### 2.2.3. Kernel Density Estimation

Kernel density estimation is often used to analyze the dynamic spatial non-equilibrium characteristic [46]. It is used to measure the spatial difference characteristic of EWP.

(15) $$f\left(x\right)=\frac{1}{nH}\overset{n}{\underset{i=1}{\sum }}K\left(\frac{E_{i}-E^{\prime }}{H}\right)$$

(16) $$K\left(x\right)=\frac{1}{\sqrt{2\pi }}e^{\left(\frac{-x^{2}}{2}\right)}$$

In Equations (15) and (16), *K(x)* represents the Gaussian kernel density; *H* is the bandwidth of regions.

#### 2.2.4. Convergence Analysis

If the EWP of a region has a σ convergence, it means that the deviation of this region’s EWP from its mean in the time series tends to decrease year by year [22].

(17) $$\sigma =\sqrt{\frac{\sum_{i}^{m}{\left(E-E^{\prime }\right)}^{2}}{n^{\prime }}}/E^{\prime }$$

In Equation (17), *n’* represents the number of cities in the region.

*β* convergence analysis. If the initial EWP of regions *A* and *B* is *a* and *b* (*a* < *b*), and the growth rate of EWP of region *A* is greater than that of region *B* over time, then region *A* has a *β* convergence [22].

(18) $$\ln\left(E_{i,t+1}/E_{i,t}\right)=\alpha +\beta \ln E_{i,t}+\overset{n}{\underset{k=1}{\sum }}\theta_{k}X_{k,i,t}+\varepsilon_{i,t}$$

(19) s=−ln(1+β)/T;t=ln2/s

where *E* represents the EWP; *i* and *t* represent city *i* and time *t*; *X* is the control variable; *s* is the convergence rate; *t* is the half-life cycle, namely, the time required to close the EWP gap. If *θ_k_* = 0, it is an absolute *β* convergence model; if not, it is a conditional *β* convergence model. The control variables include industrial structure, science and technology innovation, foreign investment, urbanization, government intervention, finance development, and environmental regulation.

If there is a spatial correlation in EWP, the spatial factor is added to the *β* convergence analysis, which can avoid biased conclusions due to neglecting spatial spillover effects.

(20) $$\ln\left(E_{i,t+1}/E_{i,t}\right)=\alpha +\beta \ln E_{i,t}+\rho \sum_{i=1}^{n}w_{ij}\ln\left(E_{i,t+1}/E_{i,t}\right)+\varphi \sum_{i=1}^{n}w_{ij}\ln E_{i,t}\;+\sum_{i=1,k=1}^{n}\theta_{k}X_{k,i,t}+\sum_{i=1}^{k=1}w_{ij}\gamma_{k}X_{k,i,t}+\varepsilon_{i,t}$$

(21) $$\varepsilon_{i,t}=\tau \sum_{i=1}^{n}w_{ij}\varepsilon_{i,t}+v_{i,t}$$

where *w_ij_* represents the spatial weight matrix. If *τ* = 0, it is the spatial Durbin model. If *τ* = 0 and *φ* = *γ* = 0, it is the spatial Lagged model. If *ρ* = 0 and *φ* = *γ* = 0, it is the spatial Error model.

#### 2.2.5. Spatial Durbin Model

The spatial econometric model examines the influential mechanism between the dependent and independent variables after considering the spatial correlation [2]. This paper initially sets the spatial Durbin model as the base model to test the spatial effects on EWP.

(22) $$\ln E_{i,t}=\rho_{0}+\rho_{1}\left(W\ln E_{i,t}\right)+\rho_{2}\left(\ln X_{i,t}\right)+\rho_{3}\left(W\ln X_{i,t}\right)+\mu_{i}+\delta_{t}+\varepsilon_{i,t}$$

In Equation (22), *W* represents the spatial weight matrix.

### 2.3. Data Sources

This paper constructs dynamic equilibrium panel data for 287 cities from 2000 to 2020 as the research subjects. According to the administrative division of the three regions of East, Central, and West, there are 121 cities in the East, 80 cities in the Central area, and 86 cities in the West. The selection of cities consists of two main aspects. First, the original number of cities is chosen based on the administrative division of Chinese cities. Second, considering the completeness of the data available on the long time series of the city dimension, the paper refers to the data in the China City Statistical Yearbook and the statistical annual reports of each city to determine the final list of cities. Due to a large number of cities, it is not convenient to show them in the main body of the paper, so they are placed in the Supplementary Materials.

The specific data are mainly from the China Statistical Yearbook, China City Statistical Yearbook, China Energy Statistical Yearbook, China Environmental Statistical Yearbook, China Health Statistical Yearbook, and the annual government work reports of cities in the corresponding years. The economic indicators are deflated using 2010 as the base period to eliminate measurement errors due to inflation. Indicators about foreign investment are first converted according to the exchange rate between the U.S. dollar and the Chinese yuan, and then deflated.

## 3. Empirical Analysis

### 3.1. Spatial and Temporal Characteristics of EWP

**Figure 1:**
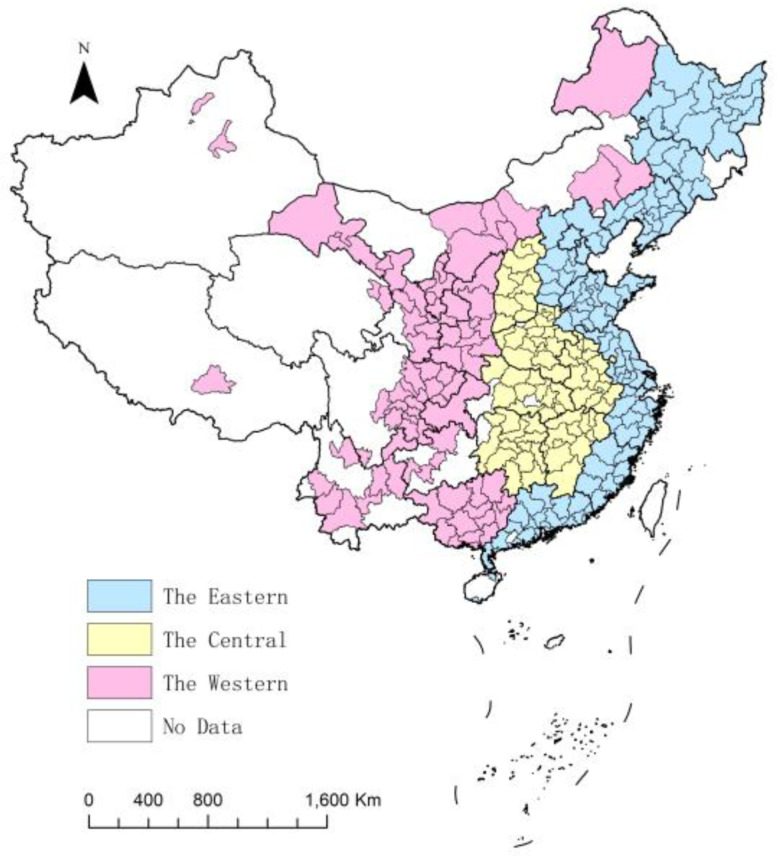
Division of regions in China.

Based on the Super-SBM DEA model, this paper estimates the EWP of 287 Chinese cities from 2000 to 2020. Meanwhile, from the four regions of national, eastern, central and western, a multi-regional EWP analysis is carried out (Figure 1).

**Figure 2:**
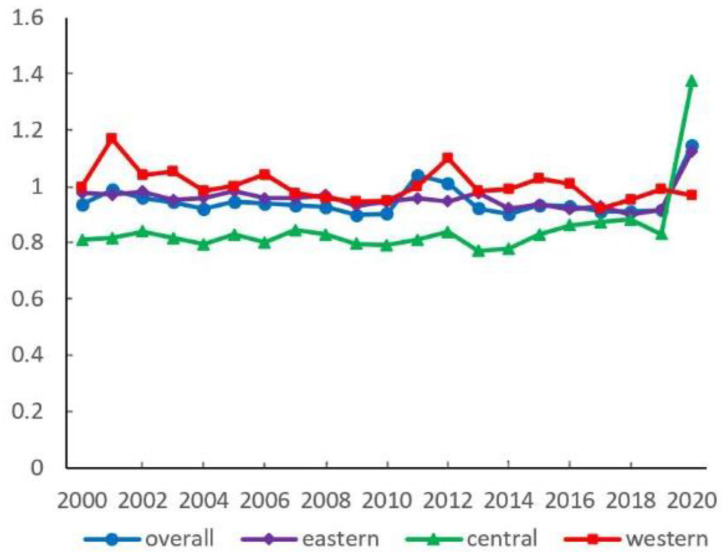
Urban EWP from 2000 to 2020.

From the national average level, urban EWP first shows a decreasing and then an increasing evolutionary trend. From 2000 to 2019, it decreases from 0.937 to 0.917, and it increases significantly to 1.149 in 2020 (Figure 2). In the time series, in general, urban EWP shows a trend of first gradually declining and then rapidly improving in eastern regions, which is similar to the national level, while the central average level is characterized by fluctuating development in the early stage and gradual improvement in the later stage. First, there is a gradual trend of decline and then improvement of EWP in the west. The enhancement of EWP is probably mainly due to the deepening of the Western Development Strategy after 2010. This regional development strategy proposes clear elements related to protecting the environment, stabilizing the economy, and enhancing social welfare.

**Figure 3:**
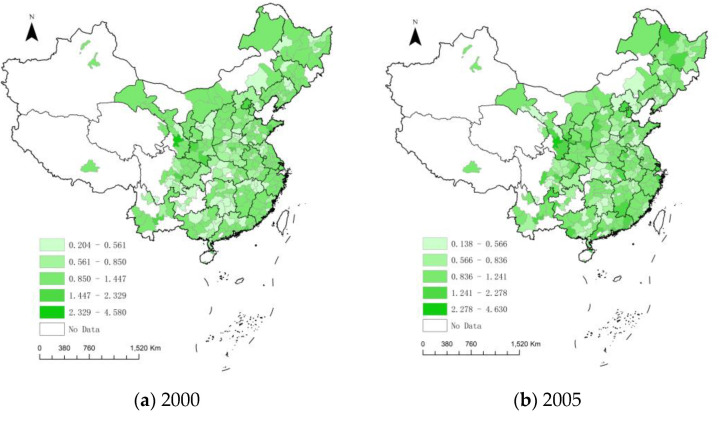

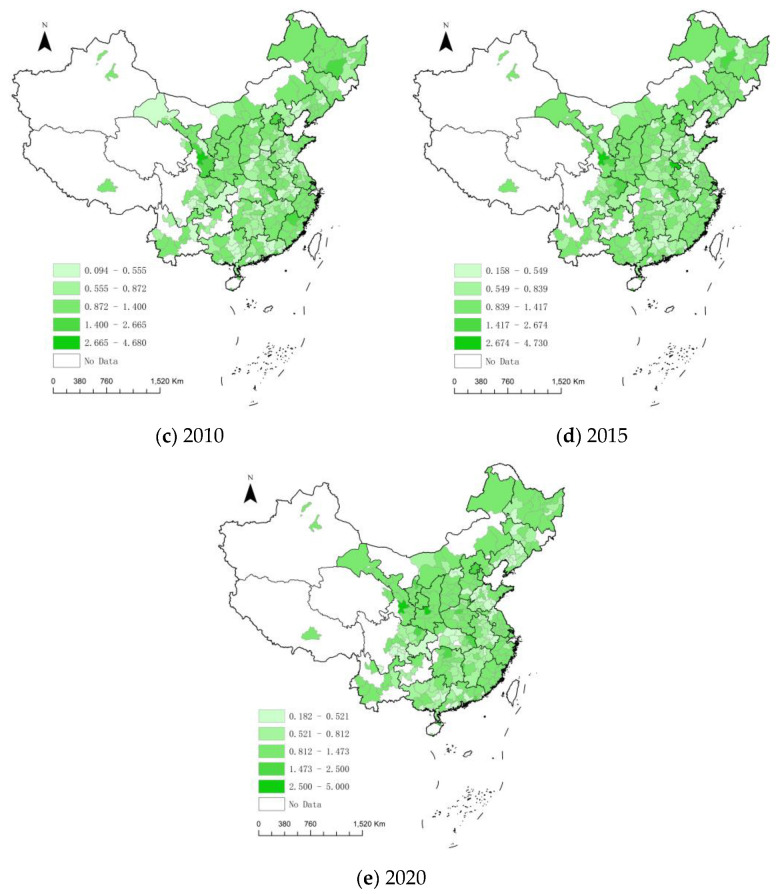
The dynamic evolution of urban EWP.

From the average level of regional dimensions, the average urban EWP of the western region is 1.004, the eastern is the second highest with 0.959, and the central region has the lowest value with 0.831. That indicates that the western region has the best-coordinated economic and environmental development. The EWP gap between the western and the central is the largest from 2000 to 2020. In total, there is a spatial distribution pattern of “high in the eastern and western, low in the central”. In each spatial dimension, cities with high EWP values are mainly provincial capitals or regional core cities in the early stage, and most of them are not spatially contiguous. However, with the elapse of time, urban EWP shows a spreading tendency from the core city to the neighboring region, with a spatial correlation in geographic space (Figure 3).

### 3.2. Regional Differences and Sources of Urban EWP

Based on the spatiotemporal evolution of urban EWP, there is an obvious spatial non-equilibrium. We further employ the Dagum Gini decomposition method to reveal the regional differences and sources of urban EWP for different spatial dimensions.

**Figure 4:**
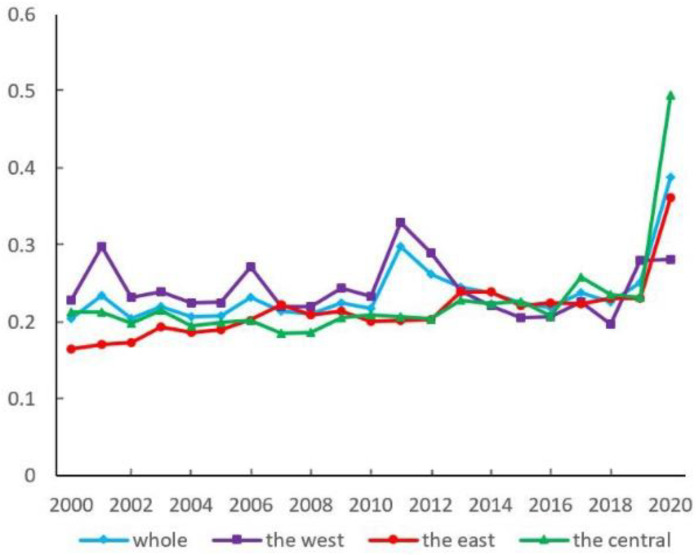
Intra-regional differences of urban EWP in each regional dimension.

As shown in Figure 4, at the overall level during the period 2000 to 2020, the intra-regional difference gradually increases in fluctuation with a value from 0.204 to 0.389. In the eastern region, the index gradually increases from 0.165 to 0.363. The central region is characterized by a trend of first decreasing and then increasing fluctuation. Western cities show a long-term fluctuating development trend, with a relatively stable period before 2010 and a gradual increase in the fluctuation period after 2010.

**Figure 5:**
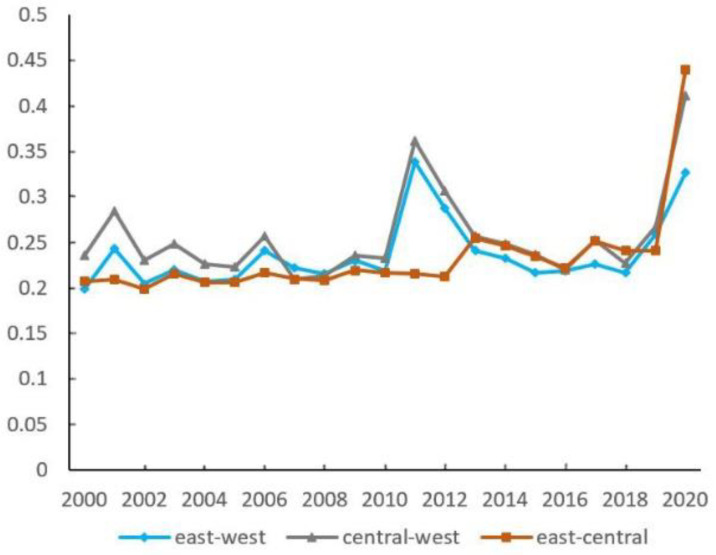
Inter-regional differences of urban EWP.

The urban EWP differences in “East-West” and “Central-West” both remain stable, fluctuating between 0.20 and 0.25. However, there is a significant trend of increase after 2019 (Figure 5). The “East-Central” difference shows a slow growth trend. The inter-regional differences rank is as follows: before 2013, “Central-West” > “East-West” > “East-Central”; after 2013, “East-Central” has the largest difference. Compared with others, eastern cities have superior development conditions to improve the efficiency of optimal resource allocation, promote industrial optimization and upgrading, enhance total factor productivity, and alleviate the overload of environmental carrying capacity by taking advantage of agglomeration and scale and encouraging green technology innovation.

**Figure 6:**
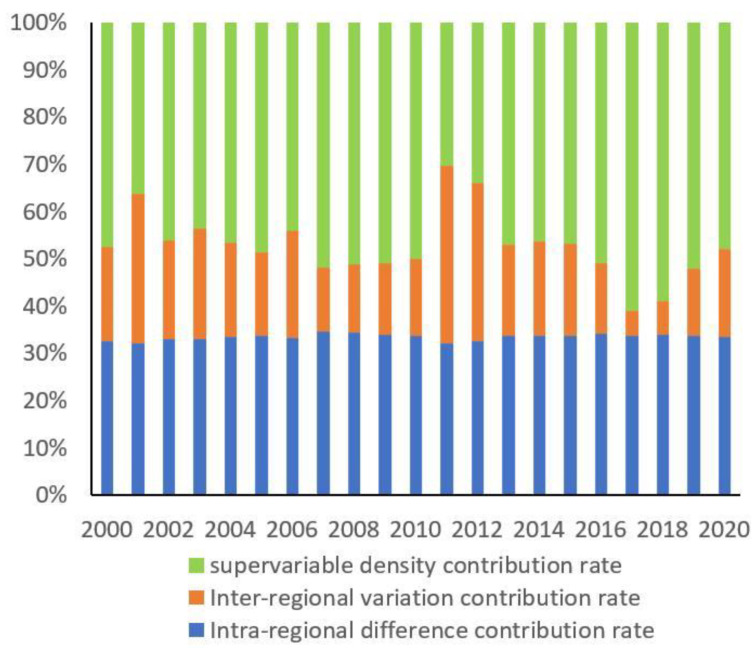
Percentages of regional difference sources of urban EWP.

In the sources of regional difference, the contribution of supervariable density is the largest at about 50%, and it has an increasing tendency (Figure 6). The contribution of intra-regional difference remains around 30% in general, which is higher than the inter-regional difference.

### 3.3. Absolute Differences Distribution of Urban EWP

The Dagum Gini coefficient decomposition model characterizes the relative differences but cannot intuitively portray the dynamic evolution of the absolute differences. This paper further employs the kernel density to examine the absolute differences distribution of urban EWP.

**Figure 7:**
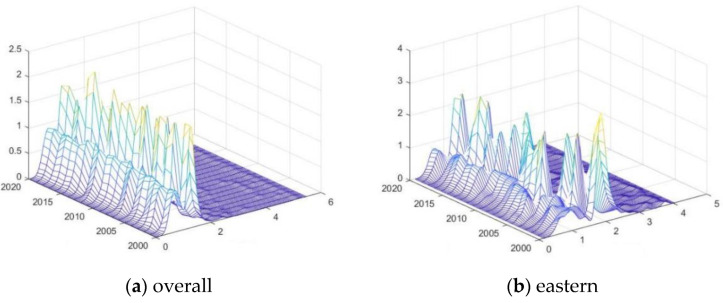

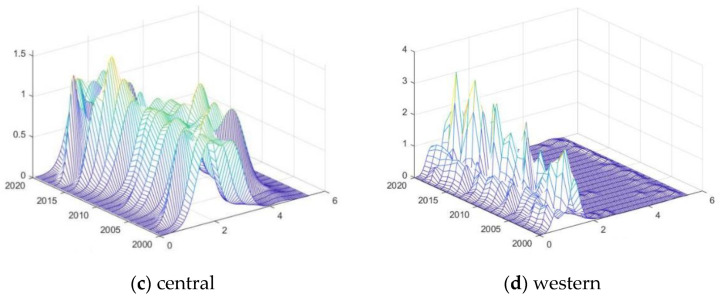
Three-dimensional kernel density analysis of urban EWP in each regional dimension. Note: the darker the color, the smaller the difference between the data.

For distribution location, urban EWP in the overall, eastern, and western regions all show a leftward first and then a rightward shifting trend, while the central region first shows a relatively stable shift, followed by a rightward shift, which is consistent with the time series evolution in the previous section (Figure 7). For the distribution pattern, in the national and eastern dimensions, the distribution curve’s main peak value shows a slowly decreasing trend in general, which indicates a trend of differences expansion. The main peak in the central region first slowly increases and then gradually decreases, indicating that the difference first narrows and then widens. The western region’s value does not change much in the early period and has an increasing trend in fluctuation in the later stage, indicating that the gap decreases later. For distribution extension, generally, there is no significant tail-lengthening phenomenon, indicating that the EWP is relatively concentrated and the gap between the high EWP and the mean level is not significant. For the polarization trend, the distribution curves all have a main peak and a side peak, indicating that there is a significant polarization effect in urban EWP.

### 3.4. Spatio-Temporal Convergence Analysis

#### 3.4.1. σ Convergence Analysis

**Figure 8:**
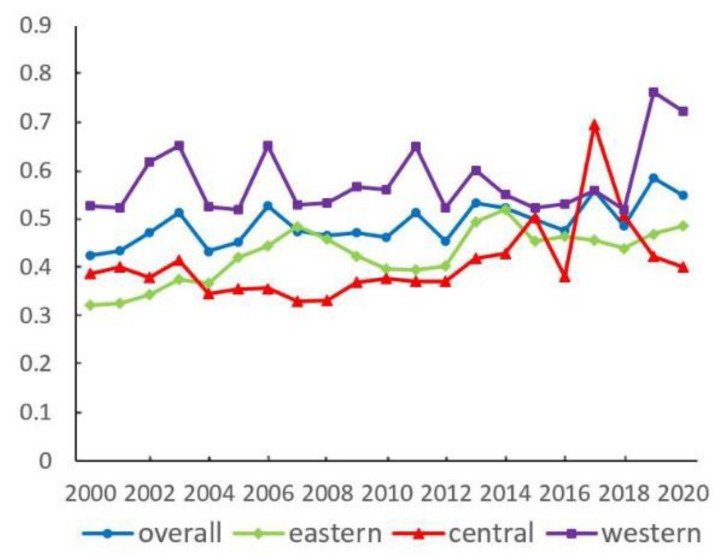
σ convergence characteristic of urban EWP in each regional dimension.

In each regional dimension, the σ convergence coefficient of urban EWP shows a roughly increasing trend in the time series (Figure 8). There is no σ-convergence characteristic.

Specifically, except for a few years, the central region has the smallest convergence coefficient, and the western region has the largest convergence coefficient. There is a certain cross-lifting feature in the convergence coefficients for the whole region and the eastern region. From 2000 to 2020, the growth values of the convergence coefficients for the overall, eastern, central, and western regions are 0.12, 0.16, 0.01, and 0.20, with growth rates of 29.34%, 50.84%, 3.54%, and 37.33%.

#### 3.4.2. *β* Convergence Analysis

As shown in Table 2, the global Moran index of urban EWP was significantly positive in the national dimension from 2000 to 2020. Therefore, urban EWP has a strong spatial correlation. Considering the spatial correlation, this paper constructs the spatial *β* convergence model.

First, the traditional *β* convergence model is constructed. Then, the spatial absolute *β*-convergence and spatial conditional *β*-convergence characteristics are analyzed.

The specific spatial econometric model type is judged by LM, Hausman, and LR tests. The results indicate that the spatial Durbin model (SDM) should be selected. The geographic neighborhood weight matrix is used as the spatial weights.

The absolute *β* convergence coefficients for each regional dimension are negative and reach the 1% significance level, regardless of whether the spatial factors are considered (Table 3). Therefore, urban EWP has absolute *β* convergence and spatial absolute *β* convergence. The converge speed is the fastest in the western region, and the eastern region is the slowest.

The conditional *β* convergence coefficients are all negative at the 1% significance level, regardless of whether the spatial influence is considered. Therefore, urban EWP has conditional *β* convergence and spatial conditional *β* convergence in each region (Table 4). The coefficient of absolute conditional *β* convergence is higher than absolute *β* convergence. Thus, the convergence speed is accelerated after considering the control variables.

The absolute coefficients of absolute *β* convergence and conditional *β* convergence obtained by the spatial Durbin model are larger than the coefficients measured by the traditional econometric model. Therefore, the convergence speed is improved after considering the spatial correlation of EWP.

### 3.5. Influential Mechanisms

According to the above analysis, it is known that urban EWP shows a strong spatial correlation. The spatial Durbin model with fixed effects is chosen to analyze the driving forces of urban EWP.

For the national dimension, the spatial autocorrelation coefficient *ρ* for EWP is significant at the 1% level. Therefore, the EWP’s spatial correlation is positive and significant (Table 5). The impact coefficients of industrial structure, foreign investment, urbanization, government intervention, and environmental regulation are −0.414, −0.010, −0.094, −0.053 and −0.039. These factors all have negative effects on EWP at the 10% significance level. When scientific and technological progress, and finance development increase by 1%, urban EWP increases by 0.066% and 0.183%, respectively. These two factors show a positive relationship with urban EWP at the 1% significance level.

In spatial effects, the spatial spillover effects of industrial structure, urbanization, finance development, and environmental regulation are all significantly positive. Government intervention shows a positive but insignificant effect. This suggests that increasing these factors will have a facilitating effect on the urban EWP of the surrounding regions. The spatial spillover effects of technological progress and foreign investment are both negative and insignificant.

Further, the spatial effects are decomposed into direct effect and indirect effect by partial differential methods. For the direct effect, industrial structure, foreign investment, urbanization, government intervention, and environmental regulation all have negative direct effects on local EWP, while science and technology and finance development show positive correlations, which are consistent with the above-mentioned influence directions. The indirect effects of industrial structure, urbanization, government intervention, finance development, and environmental regulation are all positive, while the indirect effects of science and technology and foreign investment are negative. This suggests that neighboring cities contribute to the EWP of local cities through these factors.

Compared with the national dimension, foreign investment in the eastern region plays the facilitating role. The spatial spillover effects of science and technology innovation and foreign investment are positive. The spatial spillover effects of urbanization, finance development, and environmental regulation are negative. In the central region, science and technology innovation and foreign investment play negative and positive roles, respectively, compared to the whole region. The spatial spillover effects of urbanization and finance development are both negative. In the west, the regression coefficients and spatial spillover effects are basically consistent with the evolutionary characteristics of the whole dimension, except for government intervention and environmental regulation (Table 6).

## 4. Discussion

### 4.1. About the Evolutionary Features of Urban EWP in National Dimension

With 2019 as the turning point, the mean urban EWP of the whole region has an opposite evolutionary trend. For a considerable period of time in the past, urban development focused only on economic speed, and thus, adopted a crude industrial development model. Economic development consumes a large amount of energy and resources, which leads to excessive emissions of pollutants to the environment [47]. Further, the awareness of environmental protection and environmental governance in government and society is relatively weak. Therefore, the eco-friendly development model has been increasingly emphasized. It is recognized that environmental quality and social welfare are as important as economic growth [48]. In the available studies, the overall EWP shows a decreasing trend in the time series [2,22]. In these studies, the time series of the study data are all before 2019. Therefore, they are generally consistent with the findings of this paper.

### 4.2. About the Regional Difference Features of Urban EWP in Different Regions

In the eastern region, the polarizing effect of urban EWP has always been evident. The core cities, represented by Beijing, Shanghai, Guangzhou, and Shenzhen, have continuously attracted labor, capital, and technology from surrounding regions. This agglomeration effect and scale effect accelerates the improvement of EWP in core cities [29]. However, the others have a slower improved pace, which leads to an increased trend in regional differences.

For the central region, there is a high proportion of resource-based industries. Before 2010, resource-based industries had high economic returns, which led to high welfare and environmental burden. Therefore, the intra-regional differences of EWP have a slightly decreasing trend. However, the central region currently faces the challenges of environmental pollution, resource depletion, industrial transformation and upgrading, and an imbalance of government financial investment [18], which leads to the thread of coordinated development.

The promotion of urban EWP in the western region benefits from the Western Development Strategy. From 2000 to 2010, this was the primary stage of the Strategy. The main aim is to strengthen infrastructure and optimize ecological quality [49]. After 2010, the development direction turned towards economic quality and coordinated development. Therefore, the urban EWP is relatively stable in the early stage, but fluctuant later.

Regarding sources of regional difference, the contribution of supervariable density is the most important element. Although there is a significant spatial unevenness in EWP, it does not mean that advantage is absolute [50]. In the superior region, we cannot ignore the enhancement of cities with low EWP. In the inferior region, we can rely on the core cities to cultivate a multi-level and multi-core city network system to promote the regional enhancement of EWP.

In existing studies, most scholars analyze the spatial differences in EWP based on provincial dimensions and conclude that EWP is highest in the eastern region and lowest in the western region [22]. This differs from the findings of this paper. Based on the city dimension, urban EWP in the East and West is larger than that in the central. There are two possible reasons for this. On the one hand, the data for some western cities are incomplete. In the western region, complete and long time series data are mainly from cities with better economic and social development. This further reveals the differentiated features of EWP within provinces. This conclusion cannot be shown in the macro- and meso-spatial dimensions. On the other hand, the core cities in the western region are ranked relatively high in terms of EWP. These cities are located in the inland region, and the location advantage is not obvious. However, with the implementation of the Western Development Strategy and the subsequent gradual deepening, these cities—with the advantages of good transportation, infrastructure, and cultural resources—create green industrial forms, form regional ecological corridors, and develop into a cross-regional multi-center city hierarchy integrating economy, society, environment, and industry [18].

### 4.3. About the Influential Mechanisms and Spatial Spillover Effects of Urban EWP

Based on the above analysis, we specifically analyze the mechanism behind the impact effect. A higher proportion of secondary industry will result in an excessive emission of pollutants [51], which leads to ecological deficits. Excessive foreign investment may increase the dependence on foreign mature technology, and foreign industrial migration will lead to pollution migration problems. Urbanization may encroach on ecological land and increase the ecological recycling and dissipation burden, so scientific urban planning is necessary. The formulation and implementation of environmental policies about EWP have the characteristic of time-lag [52], and excessive government intervention may crowd out the market space, which leads to the dilemma of market failure.

In the spatial effect, the increased share of the secondary industry strengthens industrial inertia and promotes the agglomeration and scale development of the industry. This leads to concentrated emissions of pollution in the region, which indirectly reduces pollution in the surrounding areas, improves the environmental quality of neighboring areas, and optimizes their ecological welfare. Therefore, increasing the share of the secondary industry will indirectly improve the EWP of the surrounding area, and the spatial spillover effect is positive. The current financial services have inclusive advantages [53] and a radiation effect on the surrounding regions, which provides support for environmental governance and ecological value marketization. Environmental regulation is dominated by the government [54] and is an important part of government performance evaluation, which leads to competition mechanisms in neighboring cities. The spatial spillover effects of science and technology innovation and foreign investment are not obvious, probably due to the market competition and trade barriers between cities.

For different regions, the spatial effects of the influencing factors are different. In the eastern region, scientific and technological progress and foreign investment have positive spatial spillover effects on urban EWP, while in the central and western regions, there are negative spatial spillover effects. This may be due to the fact that cities in the eastern region have built a more mature cooperation system, including complementarity and collaboration among cities at the same level, and cooperation and exchange among cities at different levels. This promotes interaction among cities, reduces barriers to the flow of production factors such as capital and technology, and achieves a common improvement in EWP among regions. As for the central and western regions, the polarization of core cities in terms of capital, science and technology, resources, and talents is obvious, while the collaboration among cities is not perfect and local barriers are apparent. This is not beneficial to the spread of foreign investment and technology among cities.

In the eastern and central regions, urbanization and financial development have negative spatial spillover effects on urban EWP, while in the western region they are positive spatial spillover effects. For the eastern and central regions, both urbanization and financial systems are relatively mature. If cities in these regions expand their land for construction in a disorderly manner, it will lead to the neighboring regions following suit and accelerating the “land enclosure” behavior. In turn, this will lead to the encroachment of more ecological land, reducing the ecological carrying capacity of the surrounding cities. Likewise, if a city with a more complete financial system continues to increase its investment in finance, it will crowd out the financial development space of the surrounding regions, which is not conducive to the formation of cross-regional financial collaboration. However, in the western region, urbanization and financial development still have more room for improvement, and the overall improvement of urban EWP can be achieved by optimizing population distribution, ecological restoration, and financial inclusion among regions.

The spatial spillover effect of environmental regulation on urban EWP is negative in the eastern region, while it is positive in the central and western regions. In the eastern region, cities have well-developed systems for environmental policy formulation and implementation, and public participation in environmental governance is active. If the intensity of environmental regulation is increased, it will force polluting industries to move to neighboring cities, thus, reducing the environmental quality of the surrounding area. However, in the central and western regions, there is often bottom-up competition among governments. The enhancement of environmental regulations in one city indicates an increase in the minimum threshold of environmental entry, forcing neighboring cities to enhance their environmental regulations for the purpose of performance assessment to meet the standards.

### 4.4. Shortcomings and Future Prospects

This paper provides some empirical evidence on the spatiotemporal evolution, regional differences, and driving factors of urban EWP in China, but there are still some limitations. Because of the limited availability of city-level data, the “ecological capital” indicator is only measured in two aspects: ecological service supply and municipal facilities construction. Although this is an attempt to measure ecological value, our index of ecological resources’ market value is inadequate. In the future, we will continue to collect relevant data and improve the index system to improve the objectivity and comprehensiveness of measurement. In addition, the paper only analyzes the characteristics of urban EWP from the national, eastern, central, and western dimensions, without a more detailed division of the study area. We can select smaller regions as research objects according to relevant government development strategies, analyze the spatial differences and influencing factors, and propose targeted countermeasures and suggestions for alleviating the spatial imbalance of EWP.

## 5. Conclusions and Policy Implications

### 5.1. Conclusions

Based on the relevant data of 287 cities from 2000 to 2018, this paper estimates the urban EWP by the DEA model. The empirical analysis of urban EWP’s spatiotemporal evolution, regional differences, dynamic distribution, and driving factors is carried out by the Dagum Gini coefficient decomposition model, kernel density estimation, convergence analysis, and the spatial Durbin model. The conclusions are as follows:

(1) In national, eastern, and western regions, the urban EWP shows a trend of first decreasing and then increasing. The central region has a characteristic of first fluctuating and then improving. The spatial distribution pattern is “high urban EWP in the eastern and western regions, low in the central region”.
(2) For relative difference, the intra-regional differences of urban EWP show the increasing trend in the whole and eastern regions. The central region has a trend of first decreasing and then increasing in fluctuation, while the western region shows a fluctuating characteristic. The inter-regional differences about “East-West” and “Central-West” remain stable, while the difference between “East-Central” shows a slow growth trend. For sources of regional differences, the contribution of supervariable density is the highest, followed by intra-regional difference, and the inter-regional difference is the smallest. For absolute difference, the urban EWP has a significant polarization effect.
(3) There is no σ convergence of urban EWP. However, urban EWP has spatial absolute *β* convergence and spatial conditional *β* convergence. Compared with the traditional *β* convergence analysis, the convergence speed is improved when the spatial correlation is considered.
(4) Urban EWP has a significant spatial correlation. Industrial structure, foreign investment, urbanization, and government intervention all have significant negative effects on EWP. Science and technology progress, finance development, and environmental regulation all show significant positive effects. In space, the spatial spillover effects of industrial structure, urbanization, government intervention, finance development, and environmental regulation are positive, and they are significant except for government intervention. The spatial spillover effects of science and technology progress, and foreign investment are negative, but they are insignificant.

### 5.2. Policy Countermeasures and Recommendations

Based on the empirical analysis conclusion, this paper proposes policy countermeasures and recommendations.

(1) At present, many cities have still not yet reached a mature level of EWP, and room remains for its improvement and optimization. Therefore, we should optimize the improvement paths of urban EWP by promoting economic growth, enhancing social welfare, and reducing the ecological deficit. For this, there are two main aspects. On the one hand, we should enhance social security and public services, improve residents’ living environment, and increase residents’ sense of happiness. The specific measures could include improving the efficiency of transforming economic achievements into social welfare, increasing investment in pollution control and ecological restoration, and increasing public participation in social governance. On the other hand, we should reduce the ecological footprint by improving energy utilization structure, enhancing resource utilization, promoting scientific and technological innovation, and increasing factor productivity.
(2) Based on the spatial correlation and heterogeneity characteristics of urban EWP, we should choose differentiated upgrading strategies to promote integrated and coordinated development. In eastern and western regions, urban EWP is higher than in the central region; at the same time, they are all geographically adjacent to the central region. Therefore, core cities should fully release the radiation effect of technology transfer, industrial upgrading, and environmental governance, and drive the green technology renewal, industrial transformation, and upgrading in the central region to reduce pollution emissions and improve environmental quality. In the central region, we should take into account our actual development status and formulate strategies and guidelines that are most suitable for the current stage of development, as well as strive to catch up with the EWP of other regions. Specifically, on the one hand, this could be achieved by integrating internal resources to form scale and agglomeration advantages. On the other hand, plans for technology introduction and industrial upgrading are made according to local conditions. At the same time, in the process of optimizing the economic development model, attention should be paid to the ecological vulnerability of the region and trying to reduce the production of pollutants at the source.
(3) Based on the analysis of the influencing factors of urban EWP, due to the differences in resource endowment, economic and social development conditions, and ecological carrying capacity of different regions, the enhancement mechanisms of their urban EWP are different. Therefore, the precise identification of regional development shortcomings in EWP is important for the effective promotion of coordinated development among regions. In the field of technology, talents, information, and capital, we should focus on inter-regional communication and cooperation, promote the rapid flow of production factors, and push back technological innovation. The specific measures include breaking local protection and trade barriers and accelerating the speed of technology turnover. For foreign investment, we should reduce short-term and medium-term investments and low-end industries. We should also focus on independent innovation as well as avoid the financial risks generated by the excessive inflow of foreign capital.

## Figures and Tables

**Table 1:** The urban EWP indicator measurement system.

Category	One-Level Index	Two-Level Index	Three-Level Index
input indicator	resource consumption	energy consumption	per capita consumption of coal gas, natural gas, and liquefied petroleum gas
		water consumption	water resource per capita
		land resource consumption	urban construction land area per capita
	ecological capital	ecological service supply	green space per capita
			greening coverage of built-up areas
		ecological environment improvement	length of drainage pipes per capita
			fixed asset investment in urban municipal utilities construction per capita
output indicator	undesirable outputs	wastewater emission	industrial wastewater emission per capita
		exhaust emission	SO_2_ emission per capita
		solid waste emission	proportion of industrial solid waste not comprehensively used
	desirable outputs	resident health	number of doctors per 10,000 people
			number of hospital beds per 10,000 people
		culture and education	number of teachers at college and above per 10,000 people
			number of students in college and above per 10,000 people
			library collections owned per 10,000 people
		economic capability	GDP per capita
			deposits per capita
			fiscal budget expenditure per capita

**Table 2:** Global Moran index.

Year	I	z-Value	*p*-Value	Year	I	z-Value	*p*-Value	Year	I	z-Value	*p*-Value
2000	0.096	2.528	0.006	2007	0.305	7.851	0.000	2014	0.147	3.808	0.000
2001	0.106	2.768	0.003	2008	0.293	7.569	0.000	2015	0.093	2.431	0.008
2002	0.101	2.695	0.004	2009	0.206	5.313	0.000	2016	0.135	3.493	0.000
2003	0.079	2.154	0.016	2010	0.198	5.105	0.000	2017	0.075	2.022	0.022
2004	0.14	3.664	0.000	2011	0.093	2.454	0.007	2018	0.098	2.567	0.005
2005	0.148	3.859	0.000	2012	0.159	4.111	0.000	2019	0.083	2.212	0.013
2006	0.185	4.781	0.000	2013	0.091	2.375	0.009	2020	0.14	3.657	0.000

**Table 3:** Absolute *β* convergence results.

Variable	Whole		East		Central		West	
	Non-Space	Space	Non-Space	Space	Non-Space	Space	Non-Space	Space
*β*	−0.508 *** (−42.73)	−0.512 *** (−44.01)	−0.418 *** (−24.32)	−0.427 *** (−25.24)	−0.498 *** (−22.05)	−0.500 *** (−22.69)	−0.621 *** (−27.12)	−0.621 *** (−27.85)
ρ	-	0.097 *** (5.22)	-	0.129 *** (5.06)	-	0.059 *** (4.61)	-	0.046 *** (4.42)
s	0.034	0.034	0.026	0.027	0.033	0.033	0.046	0.046
t	20.522	20.289	26.892	26.139	21.122	21.000	15.003	15.003
N	287	287	121	121	80	80	86	86
R^2^	0.086	0.087	0.065	0.066	0.098	0.098	0.108	0.109
Log-L	-	123.667	-	255.588	-	144.120	-	160.439

Note: *** means that the statistics are significant at the levels of 1%, respectively.

**Table 4:** Conditional *β* convergence results.

Variable	Whole		East		Central		West	
	Non-Space	Space	Non-Space	Space	Non-Space	Space	Non-Space	Space
*β*	−0.516 *** (−43.14)	−0.519 *** (−44.46)	−0.439 *** (−25.15)	−0.447 *** (−26.03)	−0.525 *** (−22.80)	−0.544 *** (−23.98)	−0.630 *** (−27.40)	−0.633 *** (−28.32)
ρ	-	0.092 *** (4.89)	-	0.119 *** (4.63)	-	0.030 *** (3.81)	-	0.040 *** (3.24)
s	0.035	0.035	0.028	0.028	0.035	0.037	0.047	0.048
t	20.059	19.888	25.182	24.572	19.553	18.537	14.640	14.521
con_	Yes	Yes	Yes	Yes	Yes	Yes	Yes	Yes
N	287	287	121	121	80	80	86	86
R^2^	0.084	0.086	0.050	0.051	0.1037	0.092	0.110	0.111
Log-L	-	143.216	-	279.108	-	171.377	-	177.690

Note: *** means that the statistics are significant at the levels of 1%, respectively.

**Table 5:** Influential mechanisms and spatial spillover effects of urban EWP.

Variable	Coefficient	Variable	Spatial Spillover Effect	Variable	Direct Effect	Indirect Effect	Total Effect
lnindus	−0.414 *** (−15.68)	wlnindus	0.328 *** (8.55)	lnindus	−0.400 *** (−15.17)	0.291 *** (6.87)	−0.109 *** (−2.57)
lntech	0.066 *** (7.58)	wlntech	−0.016 (−1.27)	lntech	0.065 *** (7.96)	−0.001 (−0.08)	0.064 *** (4.47)
lnfore	−0.010 ** (−2.07)	wlnfore	−0.0002 (−0.03)	lnfore	−0.010 ** (−2.14)	−0.003 (−0.40)	−0.013 * (−1.79)
lnurban	−0.094 *** (−9.92)	wlnurban	0.044 *** (2.76)	lnurban	−0.093 *** (−10.27)	0.029 (1.56)	−0.064 *** (−3.45)
lngovern	−0.053 *** (−3.81)	wlngovern	0.034 (1.61)	lngovern	−0.051 *** (−3.90)	0.029 (1.14)	−0.02 (−0.89)
lnfinan	0.183 *** (9.61)	wlnfinan	0.093 *** (3.11)	lnfinan	0.191 *** (9.98)	0.157 *** (4.88)	0.348 *** (10.23)
lnenvir	−0.039 *** (−5.20)	wlnenvir	0.080 *** (5.26)	lnenvir	−0.035 *** (−4.48)	0.088 *** (4.66)	0.053 ** (2.48)
rho	0.210 *** (12.33)	R^2^	0.256	Log_L	419.3391		

Notes: *, **, and *** denote statistical significance at the 10%, 5%, and 1% levels, respectively. The t values are in parentheses.

**Table 6:** Spatial spillover effects of urban EWP in eastern, central, and western regions.

Variable	East	Central	West	Variable	East	Central	West
lnindus	−0.453 *** (−10.32)	−0.431 *** (−7.85)	−0.325 *** (−7.46)	wlnindus	0.134 ** (2.02)	0.519 *** (5.52)	0.337 *** (5.29)
lntech	0.066 *** (4.65)	−0.032 **(−2.09)	0.079 *** (5.11)	wlntech	0.023 (1.25)	−0.066 *** (−2.73)	−0.053 ** (−2.29)
lnfore	0.069 *** (7.34)	0.002(0.15)	−0.046 *** (−6.10)	wlnfore	0.027 ** (2.08)	−0.024 (−1.42)	−0.025 ** (−2.12)
lnurban	−0.055 *** (−3.55)	−0.134 *** (−9.20)	−0.115 *** (−6.28)	wlnurban	−0.007 (−0.28)	−0.089 *** (−2.57)	0.006 (0.20)
lngovern	−0.150 *** (−6.20)	−0.046 *(−1.71)	0.028(1.21)	wlngovern	0.026 (0.71)	0.147 *** (2.77)	0.032 (0.86)
lnfinan	0.168 *** (5.18)	0.349 *** (10.36)	0.144 *** (4.34)	wlnfinan	−0.008 (−0.18)	−0.252 *** (−4.59)	0.179 *** (3.90)
lnenvir	−0.100 *** (−8.09)	−0.025 **(−2.33)	0.018 (1.12)	wlnenvir	−0.037 (−1.64)	0.023 (0.98)	0.006 (0.20)
rho	0.118 *** (4.61)	0.078 *** (2.12)	0.182 *** (6.39)	R^2^	0.369	0.3798	0.3372
Log_L	1334.3098	658.3544	1029.6936				

Note: ***, **, * means that the statistics are significant at the levels of 1%, 5%, and 10%, respectively.

## Data Availability

The data presented in this study are available on request from the corresponding author.

## Supplementary Materials

The following supporting information can be downloaded at: https://www.mdpi.com/article/10.3390/ijerph192316271/s1, Figure S1: Results of urban EWP in 287 cities from 2000 to 2020; Table S1: List of 287 cities.
