# Supplementary material for: Dynamic Evolution, Regional Differences, and Spatial Spillover Effects of Urban Ecological Welfare Performance in China from the Perspective of Ecological Value
Source: Int J Environ Res Public Health. 2022 Dec 5;19(23):16271. doi: 10.3390/ijerph192316271 (PMC9741475; doi:10.3390/ijerph192316271)
Supplement: Supplementary file 1 [file ijerph-19-16271-s001.zip › ijerph-2042639-supplementary.pdf]

Table S1. List of 287 cities

Province	City
municipality directly under the Central Government	Beijing, Tianjin, Shanghai, Chongqing
Hebei	Shi Jiazhuang, Tangshan, Qin Huangdao, Handan, Xingtai, Baoding, Zhang Jiakou, Chengde, Cangzhou, Langfang, Hengshui
Shanxi	Taiyuan, Datong, Yangquan, Changzhi, Jincheng, Shuozhou, Jinzhong, Yuncheng, Xinzhou, Linfen, Lvliang
Inner Mongolia Autonomous Region	Hohhot, Baotou, Wuhai, Chifeng, Tongliao, Erdos, Hulun Buir, Bayan Zhuoer, Wulan Chabu
Liaoning	Shenyang, Dalian, Anshan, Fushun, Benxi, Dandong, Jinzhou, Yingkou, Fuxin, Liaoyang, Panjin, Tieling, Chaoyang, Hu Ludao
Jilin	Changchun, Jilin, Siping, Liaoyuan, Tonghua, Baishan, Songyuan, Baicheng
Hei Longjiang	Harbin, Qiqihar, Jixi, Hegang, Shuang Yashan, Daqing, Yichun, Kiamusi, Qi Taihe, Mu Danjaing, Heihe, Suihua
Jiangsu	Nanjing, Wuxi, Xuzhou, Changzhou, Suzhou, Nantong, Lian Yungang, Huaian, Yancheng, Yangzhou, Zhenjiang, Taizhou, Suqian
Zhejiang	Hangzhou, Ningbo, Wenzhou, Jiaxing, Huzhou, Shaoxing, Jinhua, Quzhou, Zhoushan, Taizhou, Lishui
Anhui	Hefei, Wuhu, Bengbu, Huainan, Ma Anshan, Huaibei, Tonglin, Anqing, Huangshan, Chuzhou, Fuyang, Suzhou, Liuan, Haozhou, Chizhou, Xuancheng
Fujian	Fuzhou, Xiamen, Putian, Sanming, Quanzhou, Zhangzhou, Nanping, Longyan, Ningde
Jiangxi	Nanchang, Jing Dezhen, Pingxiang, Jiujiang, Xinyu, Yingtan, Ganzhou, Jian, Yichun, Fuzhou, Shangrao
Shandong	Jinan, Qingdao, Zibo, Zaozhuang, Dongying, Yantai, Weifang, Jining, Taian, Weihai, Rizhao, Laiwu, Linyi, Dezhou, Liaocheng, Binzhou, Heze
Henan	Zhengzhou, Kaifeng, Luoyang, Ping Dingshan, Anyang, Hebi, Xinxiang, Jiaozuo, Puyang, Xuchang, Luohe, San Menxia, Nanyang, Shangqiu, Xinyang, Zhoukou, Zhu Madian
Hubei	Wuhan, Huangshi, Shiyan, Yichang, Xiangyang, E-Zhou, Jingmen, Xiaogan, Jingzhou, Huanggang, Xianning, Suizhou
Hunan	Changsha, Zhuzhou, Xiangtan, Hengyang, Shaoyang, Yueyang, Changde, Zhang Jiajie, Yiyang, Chenzhou, Yongzhou, Huaihua, Loudi
Guangdong	Guangzhou, Shaoguan, Shenzhen, Zhuhai, Shantou, Foshan, Jiangmen, Zhanjiang, Maoming, Zaoqing, Huizhou, Meizhou, Shanwei, Heyuan, Yangjiang, Qingyuan, Dongguan, Zhongshan,

	Chaozhou, Jieyang, Yunfu
Guangxi Zhuang Autonomous Region	Nanning, Liuzhou, Guilin, Wuzhou, Beihai, Fang Chenggang, Qinzhou, Guigang, Yulin, Baise, Hezhou, Hechi, Laibin, Chongzuo
Hainan	Haikou, Sanya
Sichuan	Chengdu, Zigong, Pan Zhihua, Luzhou, Deyang, Mianyang, Guangyuan, Suining, Neijiang, Leshan, Nanchong, Meishan, Yibin, Guang-an, Dazhou, Ya-an, Bazhong, Ziyang
Guizhou	Guiyang, Liu Panshui, Zunyi, An Shun
Yunnan	Kunming, Qujing, Yuxi, Baoshan, Shaotong, Lijiang, Puer, Lincang
Tibet Autonomous Region	Lhasa
Shaanxi	Xian, Tongchuan, Baoji, Xianyang, Weinan, Yan-an, Hanzhong, Yulin, Ankang, Shangluo
Gansu	Lanzhou, Jiayu, Jinchang, Baiyin, Tianshui, Wuwei, Zhangye, Pingliang, Jiuquan, Qingyang, Dingxi, Longnan
Qinghai	Xining, Haidong
Ningxia Hui Autonomous Region	Yinchuan, Shi Zuishan, Wuzhong, Guyuan, Zhongwei
Xinjiang Uygur Autonomous Region	Urumqi; Karamay

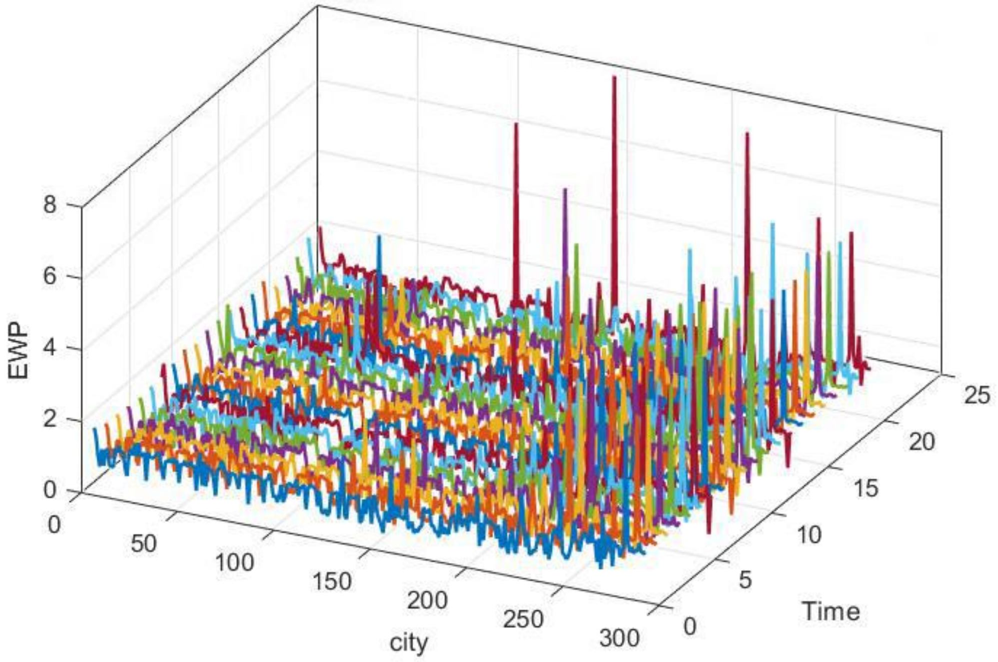

Figure S1. Results of urban EWP in 287 Cities from 2000 to 2020
